# Availability, prices and affordability of selected essential medicines in Jordan: a national survey

**DOI:** 10.1186/s12913-018-3593-9

**Published:** 2018-10-19

**Authors:** Qais Alefan, Rawan Amairi, Shoroq Tawalbeh

**Affiliations:** Department of Clinical Pharmacy, Faculty of Pharmacy, Jordan University of Science and Technology, P.O. Box 3030, Irbid, 22110 Jordan

**Keywords:** Prices, Availability, Affordability, Essential medicines, Jordan

## Abstract

**Background:**

Free access to essential medicines is a fundamental right. Governments should provide accessible and affordable medicines to people. The purpose of this study was to evaluate medicines’ prices, availability and affordability in Jordan.

**Method:**

Data was collected from 30 public sectors and 30 private sectors in 6 regions in Jordan. At each institution, the availability and prices data of 50 originator brand (OB) medicines and lowest-price generic (LPG) equivalent medicines were collected. Medicines’ prices were compared with international reference prices (IRPs) to obtain a median price ratio (MPR). Availability of medicines was determined on the day of data collection. Affordability was calculated with regards to the daily income of the lowest-paid unskilled government employee.

**Result:**

Availability of medicines in public and private sectors was 72% and 76% for LPGs, respectively. Median MPRs of procurement prices for OBs and LPGs in the public sector were 1.1 and 5.5 times the IRPs, respectively. Private sector OB medicines were priced 4.8 times higher than IRPs, whereas LPGs were 3.8 times higher. OBs cost 14% more than LPGs in private sector. The median MPRs of patient prices for LPGs in the public sector were lower than in the private sector (1.1 versus 7.6). Generally, medicines’ prices are affordable in public sector that the lowest paid unskilled government employee need less than a 1 day income to purchase the LPGs. While in private sector, the medicine prices are not affordable. For example, the treatment of hypertension either by LPGs or OBs needs more than 1 day income by lowest paid unskilled government employee.

**Conclusion:**

This study showed good availability of LPGs in the public sector. In private sector, it showed good availability of LPGs and OBs with higher patient prices. The procurement prices in the public sector were reasonable in comparison to IRPs. Policy evaluation efforts must be directed to cut medicines prices and to improve affordability in private sector.

**Electronic supplementary material:**

The online version of this article (10.1186/s12913-018-3593-9) contains supplementary material, which is available to authorized users.

## Background

Medicines play an important role in health care. They save life and reduce pain, mortality and morbidity of chronic diseases if they are accessible and affordable. Access to medicines is a universal right. However, this access is affected by several factors such as low availability, low affordability and high medicine prices. These three factors make medicines not accessible to high percentage of population and lead to harmful effects on patient’s health [1, 2]. World Health Organization (WHO) revealed that about one third of the worldwide people lack a reliable access to necessary medicines [3]. Also, about 50% of the inhabitants that live in the poorest areas of Asia and Africa lack a consistent access to essential medicines in their countries [4].

The access to needed medicines should be available and affordable for all population because it is one of their health care rights. Accordingly, the government should have policies to improve health care services and to guarantee the quality, prices, availability and affordability of important medicines [5]. WHO estimates that in low income areas 90% of citizens pay for their needed medicines out-of-pocket because of the insufficient medical and healthcare services in public sectors and most of population don’t have social insurance [6]. A study that estimated the affordability of essential medicines for treatment of chronic diseases indicated that the major obstacle that limited the access to the required medicines was the elevation in medicine prices in private sectors that couldn’t be afforded by most of people [7].

Jordan is a lower middle-income country, with a population of 9.53 million in 2016 [8]. The gross domestic product (GDP) in 2015 was US$ 37.52 billion, and GDP per capita was 3976 US dollars [9, 10]. Jordan’s health scheme is composed of many public and private sectors. Two main public sectors are the Ministry of Health (MOH) and Royal Medical Services (RMS). Other minor public sectors include university based hospitals, such as Jordan University of Science and Technology and Jordan University Hospital, and various nongovernmental programs such as United Nations Relief Works Agency (UNRWA) [11]. The health insurance plan in Jordan covers about 86% of Jordan’s population. The MOH covers 44% of the population, the RMS cover 27%, the university hospitals cover 1.3%, the private health covers 6.9% and UNRWA covers 6.8% of the population [12].

The Joint Procurement Department (JPD) was formed in 2005 with the purpose of improving the efficiency of the procurement process in the public sector [13]. The functions of JPD are to prepare and validate documents of all bids and announce them; arrange procurement procedures, participation terms, bids study method; and conclude procurement contracts [13, 14]. The supply and purchasing department of the MOH administers the storage and distribution of medicines to all MOH institutions [14, 15]. The procurement and distribution of medicines in the private sectors is done by a few large importers and wholesalers. They deliver medicines from manufacturers or suppliers and selling them to private hospitals and retail pharmacies. All manufacturers, wholesalers, importers and retail pharmacies must be registered with the JFDA [13, 15].

Jordan Food and Drug Administration (JFDA) is responsible for putting fixed national retail prices for medicines in sector, but not in the public sector. It sets a fixed profit margin between medicines manufacturers/importers and pharmacies and examines the prices after 2 years of registration and revises prices of all medicines every 5 years upon registration renewal [16].

Studies on medicines availability, prices, and affordability in Jordan are very limited. The latest study in Jordan was conducted in 2004 and was not published [17]. WHO recommends conducting such studies every 2 years to assess the impact of policy and programmatic changes on the prices of medicines. Thus, the main goal of this study was to evaluate medicine prices, availability and affordability in Jordan.

## Methods

This was a cross sectional survey that was conducted between June and October 2016. Data was collected on the medicine prices, availability and affordability from both public and private sectors in six regions in Jordan following WHO/HAI methodology [18, 19].

### Selection of medicine to be surveyed

A list of 50 medicines was included in this study (Additional file 1). The list of medicines consisted of global core list of 14 medicines to enable international comparisons; a regional core list of 16 medicines to make comparisons between countries in the same region; and a supplementary list of 20 medicines that were chosen for their local importance.

### Selection of medicine outlets

Data was collected in six cities (Amman, Irbid, Alzarqa, Ajloun, Almafraq and Jerash). The selection of cities was done according to WHO manual in this sequence: firstly, the major urban centre (Amman) was chosen. Secondly, the other five areas were selected according to: 1) every area can be arrived in a 1 day’s drive from the major urban center; 2) every area should cover population range from 100,000 to 250,000 and 3) every area should include the required number of health care facilities.

The medicine outlets in each city were chosen according to WHO method. In every area, the main public hospital was selected, then four other public institutions (e.g., hospital pharmacies, health center dispensaries) were chosen randomly provided that it can be reached within 3 hours drive from the main public hospital. Then, private sector medicine outlet (e.g., licensed pharmacy/medicine store) was chosen within 10 KM from each public medicine outlet.

### Data collection

Medicine facilities were visited to collect data about the prices and the availability of medicines. In all facilities, data was collected on the same dosage form, strength and pack size for each medicine to allow local and international comparisons to be made. For every medicine, data was collected for both products: the OB (the international brand product for the medicine) and the LPG equivalent (any product other than the originator brand that includes the same active ingredient). Price information was collected for 1) procurement prices only in public sectors; and 2) patient prices in both public and private sectors.

### Data entry

Price data was entered into the pre-programmed MS Excel® workbook according to WHO/HAI methodology. Data was entered and checked two times to guarantee the quality of data and to prevent any errors.

### Data analysis

#### Medicine prices

Price data for individual medicines was presented in two ways:

1. Median unit prices of the medicine.
2. A ratio to an international reference price (IRP) expressed as the Median Price Ratio (MPR) to facilitate international comparison.

The MPR was obtained by dividing median local price of the medicine to the IRP. This ratio was an expression of how much greater or lesser the MPR in comparison with the IRP. For example, an MPR of 2 represents that the local medicine price is twice the IRP.

Management Sciences for Health (MSH 2014) Guide was the source for the IRPs [20]. The latest IRPs that were used in this study was 2014 IRPs and all prices used were adjusted to MSH 2014 prices. The resulted data that measured procurement and patient prices was expressed as Median (mid-point) of the MPRs for medicines; 25th percentile MPR; 75th percentile MPR; Minimum MPR; and Maximum MPR.

The ideal value for MPR was used to represent acceptable local price ratios:

- Procurement prices in the public sector MPR ≤ 1
- Patient prices in the public sector MPR ≤ 1.5
- Patient prices in private pharmacies MPR ≤ 2

If the MPR for patient prices in public and private sector is twice or more the IRP then it raises a problem that the prices become unaffordable.

#### Medicine availability

Availability of each medicine was expressed as the percentage of facility availability of the required medicine on the day of data collection as follow: Absent: 0% of facilities, these medicines were not found in any facility; Low: < 50% of facilities, these medicines were difficult to found; Fairly high: 50–80% of facilities, these medicines were available in several facilities; and High: > 80% of facilities, these medicines have good availability.

#### Medicine affordability

The lowest paid unskilled government worker earns 5.83 JD (US$ 8.28) in 1 day in Jordan. Affordability was estimated by determining the required number of working days of the lowest-paid salary to unqualified workers that able them to buy standard treatment for common conditions [18]. Standard treatments for acute diseases meant to buy full courses of therapy and to buy one-month course of therapy for chronic diseases. Treatment that cost only 1 day income or less (for a 7-day supply of medicine for an acute condition or 1 month supply of medicine for chronic diseases) will be considered as affordable in public and private sectors.

## Results

In this study, 30 public institutions and 30 private retail pharmacies were accessed for data collection according to WHO/HAI methodology.

### Availability of medicines in the public and private sectors

In public sector, the mean availability of OBs was low (9%) and for LPGs was fairly high (72%). In private sector, the mean availability of OBs and LPGs were fairly high (57% and 76%, respectively) (Table 1).

**Table 1:** Availability of medicines in public and private sectors

	Public		Private	
Type of medicine	OBs	LPGs	OBs	LPGs
Availability	9%	72%	57%	76%

**Fig. 1:**
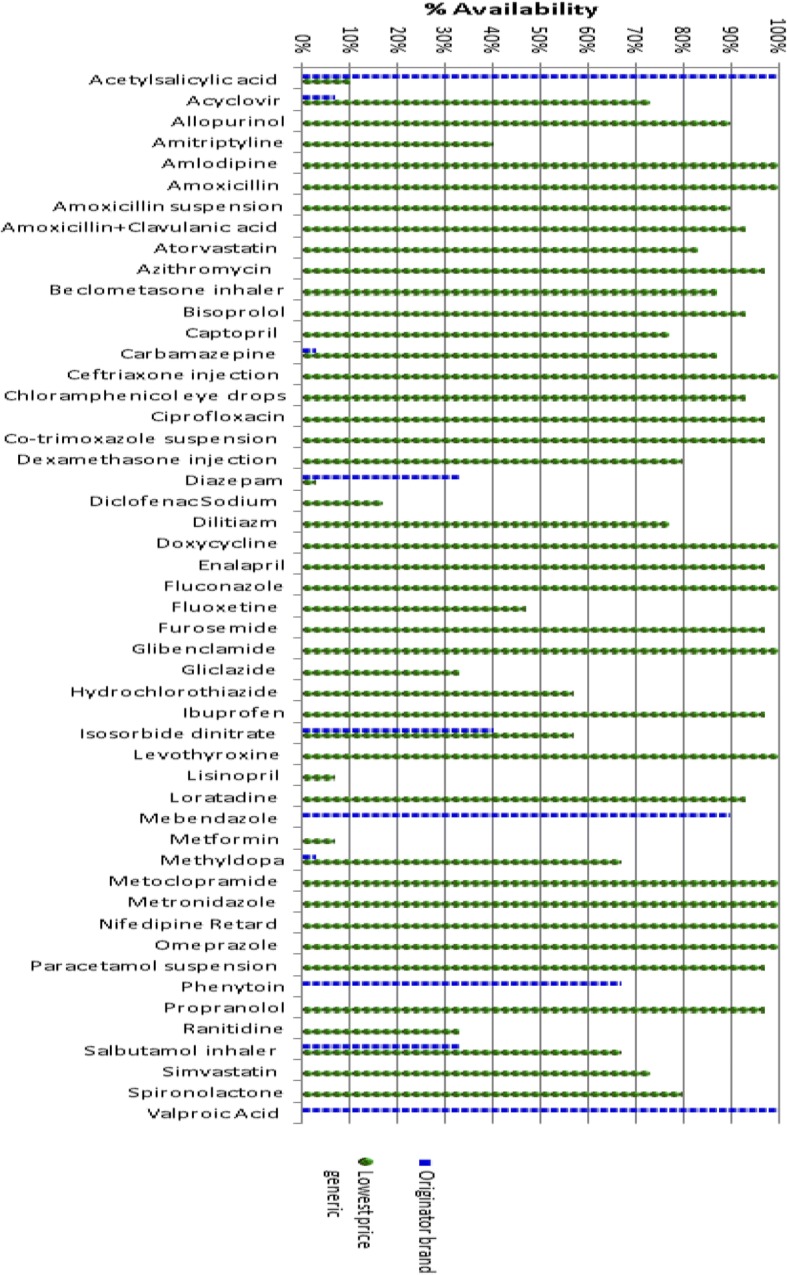
The availability of LPG and OB medicines in public sector

**Fig. 2:**
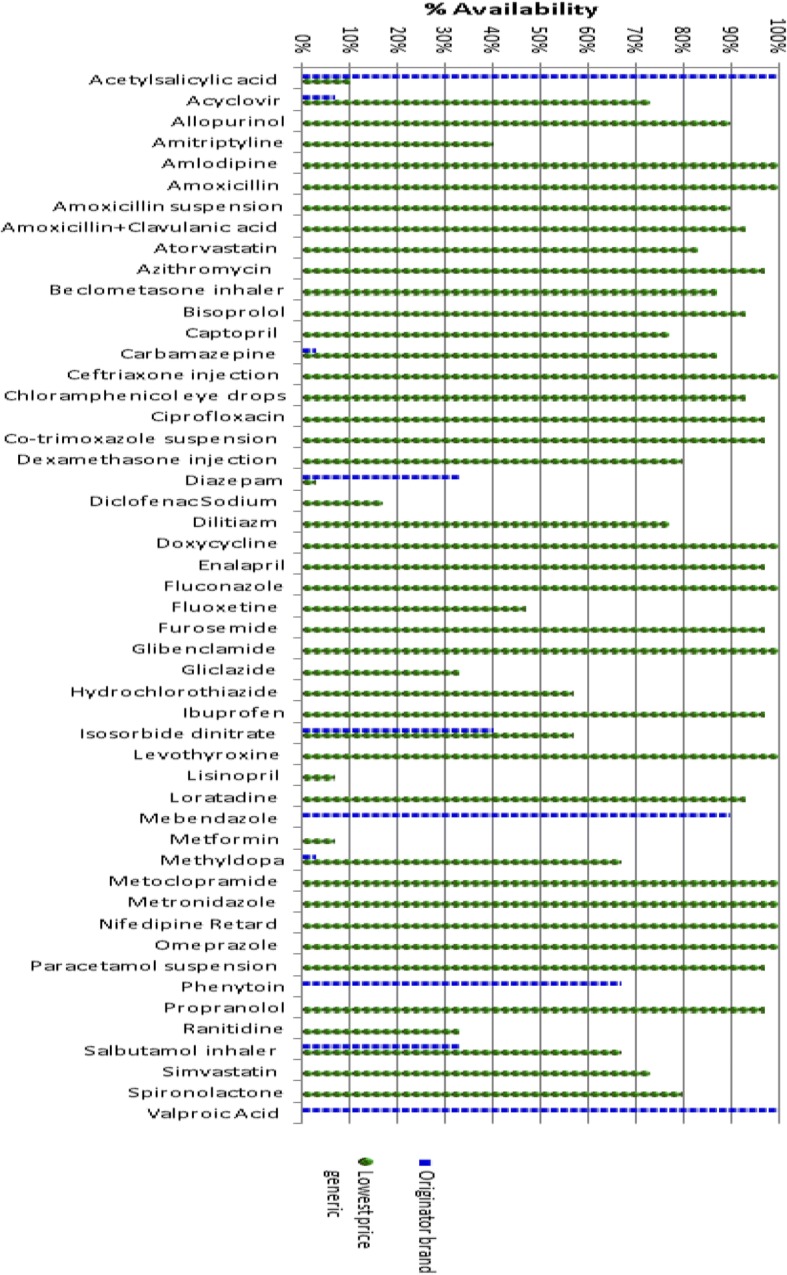
The availability of LPG and OB medicines in private sector

Table 2 expresses the mean availability of each medicine in public and private sectors. In public sector, only eight medicines’ availability were found to be low and 29 medicines were found in more than 90% of all outlets in public sector. In private sector, nearly all medicines were found in all pharmacies. However, LPG for phenytoin and the OBs for five medicines (i.e., Chloramphenicol eye drops, Enalapril, Nifedipine Retard, Paracetamol suspension, Propranolol) were not available (0%). The availability of all medicines in public and private sectors is shown in Additional file 2. Figures 1 and 2 shows the mean availability of LPGs medicines in public and private sectors.

**Table 2:** Availability of medicines in public and private sectors

Availability	Range	Public sector (*n* = 30 outlets)		Private Sector (*n* = 30 outlets)	
		OB	LPG	OB	LPG
Absent	0%	The rest of brands surveyed	Phenytoin, Valproic Acid	Chloramphenicol eye drops Enalapril Nifedipine Retard Paracetamol suspension Propranolol	Phenytoin
Low	< 50%	Acyclovir, Carbamazepine, Diazepam, Isosorbide dinitrate Methyldopa, Salbutamol inhaler	Acetylsalicylic acid, Amitriptyline, Diclofenac Sodium, Diazepam, Fluoxetine, Gliclazide, Lisinopril, Metformin, Ranitidine	Ceftriaxone injection Dexamethasone injection Fluoxetine Hydrochlorothiazide Gliclazide	Isosorbide dinitrate Methyldopa
Fairly High	50–80%	Phenytoin	Acyclovir, Captopril, Dexamethasone injection, Dilitiazm, Hydrochlorothiazide, Isosorbide dinitrate, Methyldopa, Salbutamol inhaler, Simvastatin, Spironolactone,	Acyclovir, Allopurinol Amitriptyline, Amoxicillin Amlodipine, Amoxicillin suspension, Amoxicillin+Clavulanic acid, Atorvastatin Azithromycin, Beclometasone inhaler Bisoprolol, Captopril Carbamazepine Ciprofloxacin Co-trimoxazole suspension Diazepam, Diclofenac Sodium, Dilitiazm Doxycycline, Fluconazole Glibenclamide, Isosorbide dinitrate, Ibuprofen Lisinopril, Loratadine Mebendazole, Methyldopa Metoclopramide Metronidazole, Omeprazole, Ranitidine Salbutamol inhaler Simvastatin, Spironolactone Valproic Acid	Acyclovir Amitriptyline Amlodipine Amoxicillin Amoxicillin suspension Amoxicillin+Clavulanic acid Beclometasone inhaler Bisoprolol Diazepam Diclofenac Sodium Dilitiazm Fluoxetine Gliclazide Hydrochlorothiazide Ibuprofen Levothyroxine Lisinopril Loratadine Propranolol Salbutamol inhaler Simvastatin Spironolactone Valproic Acid
High	> 80%	Acetylsalicylic acid, Mebendazole, Valproic Acid	Allopurinol, Amlodipine, Amoxicillin, Amoxicillin+Clavulanic acid, Amoxicillin suspension, Bisoprolol, Beclometasone inhaler, Azithromycin, Atorvastatin, Carbamazepine, Ceftriaxone injection, Chloramphenicol eye drops, Ciprofloxacin, Co-trimoxazole suspension, Doxycycline, Fluconazole, Glibenclamide, Furosemide, Enalapril, Ibuprofen, Levothyroxine, Loratadine, Metoclopramide, Nifedipine Retard, Omeprazole, Metronidazole, Paracetamol suspension, Valproic Acid.	Acetylsalicylic acid Furosemide Levothyroxine Metformin Phenytoin	Acetylsalicylic acid Allopurinol Amoxicillin+Clavulanic acid Atorvastatin Azithromycin Captopril Ceftriaxone injection Carbamazepine Chloramphenicol eye drops Ciprofloxacin Dexamethasone injection Co-trimoxazole suspension Diazepam Doxycycline Enalapril Fluconazole Glibenclamide Ibuprofen Metformin Mebendazole Metoclopramide Metronidazole Nifedipine Retard Paracetamol suspension Ranitidine

### Prices of medicines in the public and private sectors

#### Procurement prices in public sector

The results from procurement prices were used to find how efficiently a procurement system is working. Having the median of MPRs equal 1.00 or less indicates that the procurement system is working very efficiently and effectively. On the other hand, MPRs above 1.00 might raise concerns about purchasing efficiency [18, 21].

Public procurement prices were available for 48 out of 50 medicines studied with 3 medicines were procured as OBs and 45 as LPGs. As shown in Table 3, LPGs were purchased at 1.13 times more than the ideal value of MPRs and OBs were procured at 5.55 times more than the ideal value of MPRs. For all the medicines surveyed, there was wide variation in the MPRs of LPGs (0.15–9.06) and OBs (0.53–7.87).

**Table 3:** Public sector procurement prices as MPRs for purchased medicine^a^

Type and number of medicines	Median MPR	25th percentile MPR	75th percentile MPR	Minimum MPR	Maximum MPR
OBs (*n* = 3)	5.55	3.04	6.71	0.53	7.87
LPGs (*n* = 45)	1.13	0.65	1.56	0.15	9.06

^a^Procurement MPRs for each medicine are listed in Additional file 3

#### Patient prices in public sector (co-payment of patients)

As shown in Table 4, the MPRs of LPGs ranged from (0.06–5.3) with a median MPR equal to 1.16 in public facilities. The MPRs of OBs ranged from (0.56–8.3) with a median MPR equal to 5.8, which was 3.86 times higher than the ideal value of MPRs.

**Table 4:** Patient prices for medicines found in 4 or more public facilities ^a^

Type and number of medicines	Median MPR	25th percentile MPR	75th percentile MPR	Minimum MPR	Maximum MPR
OB (*n* = 3^b^)	5.8	3.2	7.0	0.56	8.3
LPG (*n* = 42)	1.16	0.5	1.5	0.06	5.3

^a^Patient prices as MPRs in public sector are listed in Additional file 4

^b^only 3 OBs were found in public sector

#### Patient prices in private sector (co-payment of patients)

Patient prices data were expressed as MPRs in order to facilitate comparison between LPG and OB medicines. As shown in Table 5, patient prices for OB medicines were about 4.8 times higher than the IRPs, with half of the medicines priced 2–14 times more than the ideal value of MPR. LPGs were 3.8 times more than IRPs, with half of the medicines priced 1.5–8 times more than the ideal value of MPR. For all the medicines studied, there was wide variation in the MPRs of LPGs (0.8–73.8) and OBs (0.64–146.9). The highest priced medicine for the OB was Fluconazole (MPR = 146.9) and had the highest generic price (MPR = 73.8).

**Table 5:** Patient prices for medicines found in 4 or more private facilities^a^

Type and number of medicines	Median MPR	25th percentile MPR	75th percentile MPR	Minimum MPR	Maximum MPR
OB (*n* = 45)	9.7	4.0	28	0.64	146.9
LPG (*n* = 49)	7.5	3.0	16	0.8	73.8

^a^Patient prices as MPRs in private sector are listed in Additional file 5

Table 6 shows only medicines with prices found for both types in pairs (OB and LPG). Generally, OBs cost 14% more than the LPG equivalents. The percentage of OBs that had MPR over 10 was 44.4%, while 38.8% of generics had MPR over 10 as shown in Additional file 5.

**Table 6:** Patient prices for OBs and LPGs in private pharmacies

Type of medicines (*n* = 44)	Median MPR	25th percentile MPR	75th percentile MPR	Minimum MPR	Maximum MPR
OB	8.8	3.7	28.0	0.64	146.9
LPG	7.5	3.2	17.0	0.8	73.8

#### Patient prices in public and private sectors

In order to compare prices between public and private sectors only medicines that found in both sectors were included. Data showed that, patient prices in the private sector for LPGs were 6.5 times higher than the prices in the public sector. Also, the prices of OBs in private sector were 1.6 times higher than the prices in public sector (Table 7).

**Table 7:** Median MPRs for medicines found in both the public and private sectors

Type and number of medicines in both sectors	Median MPR public sector patient prices	Median MPR private sector patient prices	% Difference private to public
OB (n = 3)	5.8	9.7	68%
LPG (n = 42)	1.16	7.6	554%

**Fig. 3:**
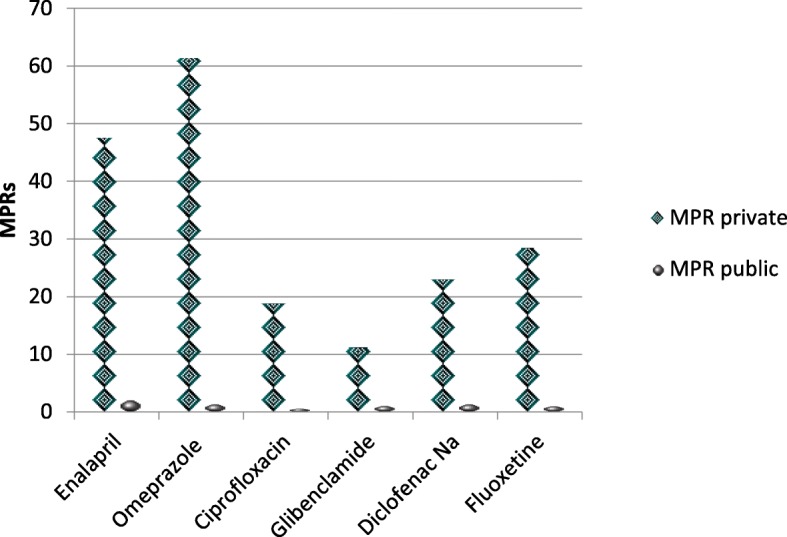
Patient prices of LPGs in public and private sectors

As shown in Fig. 3, six medicines were used to show the high differences in LPG prices in private and public sector. Patients purchasing the LPGs (Enalapril, Omperazole, Ciprofloxacin, Glibeclamide, Diclofenac sodium and Fluoxetine) from the private sector should pay 19, 39, 26, 9, 14, 23 times, respectively more than the prices of the public sector.

#### Affordability of standard treatment regimens

As shown in Table 8, the standard treatment for all conditions in public sector cost 1 day income or less of the lowest paid unskilled government worker. In the private sectors, the standard treatment of conditions such as asthma, diabetes, depression, adult respiratory infection treated with OB or LPG of amoxicillin, pediatric respiratory infection, and pain/inflammation for children, anxiety and epilepsy cost only 1 day income or less of the lowest paid unskilled government worker. However, the standard treatment of conditions like hypertension, Hypercholesterolemia, arthritis, adult respiratory infection treated either by LPG or OB of ciprofloxacin or ceftriaxone injection and ulcer need more than 1 day income of the lowest paid unskilled government worker.

**Table 8:** Number of days’ wages of the lowest paid unskilled government worker required to pay for standard treatments

Condition	Drug name	Dosage and Duration	Day’s wages to pay for treatment		
			LPG public sector	LPG private sector	OB private sector
Asthma	Salbutamol Inhaler	1 inhaler (200 doses) As needed	0.3	0.3	0.6
Diabetes	Metformin	500 mg three times daily X 30 days	Not found	0.6	0.7
Hypertension	Bisoprolol	5 mg twice daily X 30 days	0.1	1.6	1.5
Hypertension	Captopril	25 mg twice daily X 30 days	0.1	1.6	2.3
Hypercholesterolemia	Simvastatin	20 mg once daily X 30 days	0.1	1.2	1.5
Depression	Amitriptyline	25 mg three times daily X 30 days	0.3	0.5	1.0
Arthritis	Diclofenac	50 mg twice daily X 30 days	0.1	1.1	1.5
Ulcer (duodenal)	Omeprazole	20 mg once daily X 30 days	0.1	4.0	1.9
Ulcer (peptic)	Ranitidine	150 mg twice daily x 30 days	Not found	1.2	1.5
Adult respiratory infection	Amoxicillin	500 mg three times daily ×7 days	0.1	0.6	0.8
Pediatric respiratory infection	Co-trimoxazole suspension	8 + 40 mg/ml 5 mL twice daily × 7 days	0.0	0.1	0.3
Adult respiratory infection	Ciprofloxacin	500 mg twice daily × 7 days	0.1	1.4	4.4
Adult respiratory infection	Ceftriaxone injection	1 g/vial 1vial × 1 day	0.1	2.2	3.3
Anxiety	Diazepam	5 mg daily ×7 days	Not found	0.2	0.0
Pain/ inflammation children	Paracetamol suspension	24 mg/ml 5 mL three times daily × 7 days	0.0	0.	Not found
Epilepsy	Carbamazepine	200 mg twice daily x 30 days	0.2	0.7	0.9

**Fig. 4:**
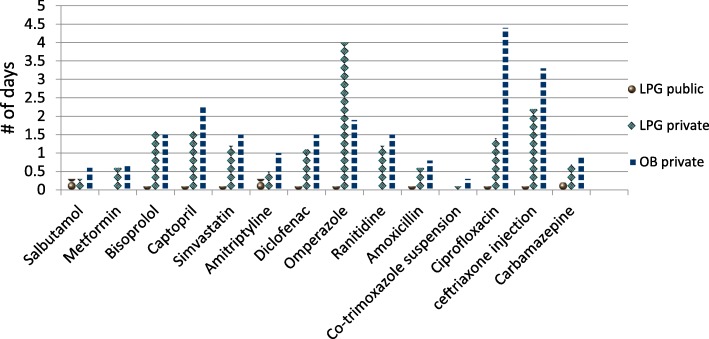
Affordability of selected medicines in public and private sectors

In Fig. 4, these treatment regimens were used in order to compare the difference in number of days needed to purchase medicines from public and private sectors. The results showed that purchasing medicines for these treatment regimens in private sector need more days of wage than purchasing them from public sector.

### International prices comparisons

#### Procurement prices in public sector

Table 9 illustrates Jordan public sector procurement price MPRs for seven medicines (Amoxicillin, amitriptyline, captopril, diclofenac sodium, diazepam, salbutamol inhaler and metformin) in comparison to six other countries (Egypt, Saudi Arabia, Sudan, Lebanon, Oman and India). The prices were obtained from HAI website and were adjusted to MSH 2014 reference prices [19, 20]. The result showed that Jordan paid the lowest prices for amoxicillin. However, Jordan paid the highest price for Amitriptyline. The price of captopril in Jordan is similar to the prices in Saudi Arabia, Egypt and Oman. While, it was lower than the prices in Sudan and Lebanon. Oman paid the highest prices for diazepam and salbutamol inhaler, and Sudan paid the highest price for metformin. However, India paid the lowest prices for all LPG medicines except for amoxicillin.

**Table 9:** MPRs of procurement prices in public sector for OBs and LPGs

Medicine name	Jordan		Egypt		Saudi		Sudan		Lebanon		Oman		India	
	OB	LPG	OB	LPG	OB	LPG	OB	LPG	OB	LPG	OB	LPG	OB	LPG
Amoxicillin		0.98						1.50		1.40				1.10
Amitriptyline		3.17		2.08				2.58		1.80	3.6			0.09
Captopril		0.65		0.79	11.9	0.58		3.60		2.08		0.50		
Diclofenac Na		1.48				3.30		19.4		7.90		1.00		0.29
Diazepam		3.62						2.30				6.80		
Salbutamol inhaler		1.08	0.76	0.48	0.67			1.00		0.86		5.70		0.57
Metformin		0.89		0.41	3.3	1.00		1.50				0.40		0.29

#### Patient prices in private sector

Table 10 illustrates patient price MPRs in the private sector for seven medicines in Jordan and six other countries (Egypt, Saudi Arabia, Sudan, Lebanon, Oman and India). The result showed that the patient prices of LPGs for amoxicillin, captopril, diclofenac sodium, diazepam and metformin were the highest in Jordan. However, the price of LPGs such as salbutamol inhaler in Jordan was comparable to other countries with the highest price was in Oman (MPR 4.8). Also, the price of amitriptyline LPG in Jordan was lower than Lebanon and Sudan but higher than the prices in Egypt and India. Also, the patient prices for OBs of amoxicillin and diazepam in Jordan were lower than in the other countries. The patient prices for OBs of captopril and metformin in Jordan were higher than the prices in Egypt and Saudi Arabia. The OB of salbutamol inhaler in Jordan was lower than the prices in Lebanon and Oman but it was higher than the prices in Egypt, Saudi Arabia, India and Sudan. Also, in India the OB of amitriptyline and diclofenac sodium prices was lower than all countries.

**Table 10:** MPRs of patient prices in private sector for OBs and LPGs

Medicine name	Jordan		Egypt		Saudi		Sudan		Lebanon		Oman		India	
	OB	LPG	OB	LPG	OB	LPG	OB	LPG	OB	LPG	OB	LPG	OB	LPG
Amoxicillin	9.8	7.5						3.09	14	5.4	11	4.9		6.7
Amitriptyline	12.1	6.2		2.4				6.38		8.75	17.9		10.4	5.8
Captopril	22.4	15.9	5.3	3.3	17.9	12.9		7.2	22.6	11.9				7.2
Diclofenac Na	29.9	23			65.7	20.4		22.4	74.5	1.6	11.3	16.4	10.6	5.2
Diazepam	5.98	23.8		5.1				4.7	25.8		24			
Salbutamol inhaler	2.35	1.2	0.9	0.85	2	1.6	2	1.2	2.6	1.2	5.8	4.8	1	1
Metformin	3.87	3.2	2.6	1.7	5.2	2	5	2.7			4.4	2.6		1.5

## Discussion

This study evaluated medicine prices, availability and affordability to population and the prices were accurately compared to international prices using WHO/HAI manual [18, 19].

### Availability of surveyed medicines

Medicines should be sufficiently available to patients in public and private sector in order to get their treatments appropriately and to accomplish the ultimate goal of improving their quality of life [1]. This study found that, in public sector, the availability of LPG medicines was high but not for OB medicines. This demonstrates effective generic policy implementation by the government in the public sector. While, in private sector, the availability of LPG and OB medicines was fairly high. Such results also suggest that patients’ populations covered by the public sector are mainly treated with LPG medicines. However, they can get the OB medicines from private sectors if they choose to be treat with OB medicines. These results were similar to the findings of a study that was conducted in Sri Lanka and showed a fairly high availability of LPG medicines in public and private sectors (58% and 74.4%, respectively) [22]. While, another study found low availability of LPGs in public sector [23]. Also, another study which was done in six low and middle income countries (Bangladesh, Brazil, Malawi, Nepal, Pakistan and Sri Lanka) demonstrated a considerably lower total availability of medicines in public sectors in all countries when compared to private sectors [7]. On the other hand, a study conducted in rural areas of China showed low availability of LPGs in the public and private sectors (39% and 44%, respectively) [24]. Overall, Jordan healthcare system is doing a fairly good job in making drugs available to patients in both public and private sectors.

### Prices of surveyed medicines

The study showed that in the public sector, the procurement prices of LPGs were 13% higher than the IRPs. While, procurement prices of OBs were 5.5 times more than the IRPs. This indicates that the public sector procurement prices of LPGs and OBs are higher than the IRPs. These differences propose considerable variation in price mark-ups between medicines. Therefore, using of IRPs as a yardstick to guarantee lower procurement prices in the public sector should be encouraged.

Similarly, a study was conducted in China showed that the procurement prices of OBs were higher than the procurement prices of LPGs [24]. Thus, procuring generic medicines help in making efficient and cost-saving procurement process [7]. Another study which was done in Sri Lanka showed a slightly lower procurement prices than the IRPs for LPGs in public sector (MPR 0.82) which indicates an efficient procurement process [25]. Similarly, studies in India and Swaziland also revealed cost saving procurements for LPGs and that MPRs were (0.68 and 0.96, respectively) in public sector [26, 27].

In general, LPGs in public sector are sold to patients at reasonable prices compared to IRPs (MPR 1.16) and within the range recommended by WHO (MPR ≤ 1.5). However, OBs are sold to patients at much higher prices (MPR 5.8) that are almost four times higher than the IRPs. This indicate that LPGs sold to patients at prices lower than OBs.

Private sector patient prices for OBs and LPGs were almost five times and four times higher than IRPs, respectively. Thus, prices of OBs and LPGs in private pharmacies are considered high. Also, the patients are paying more to buy OBs (MPR 9.7) when compared to LPGs (MPR 7.5) in private sector. This result is comparable to the result of a study, which was done in Swaziland and showed that the patient prices in private sector for OBs cost 4.7 times more than the prices of LPGs [26]. Also, another study showed that in private pharmacies, the patient prices for OBs and LPGs were 16 and 6.6 times higher than IRPs, respectively [23].

In this study, when patient prices in public and private sector were compared, the results showed that patient prices for LPGs and OBs in private sector were 554% and 68% higher than patient prices in public sector, respectively. Therefore, the public sector in Jordan is able to offer more affordable patient prices compared to the private sector. Consistent with this results, a study showed that patient prices of LPGs in private sector (MPR 7.57) were 6.5 times higher than the prices in the public sector (MPR 1.16) [26]. While, in another study, the patient prices for LPGs in private sector were lesser than in the public sector (MPR 1.82 and 3.54, respectively) [27].

### Affordability of surveyed medicines

Affordability was calculated as the number of days the lowest paid unskilled government worker would have to work to pay for one treatment course for an acute condition or 1 month‘s supply of medicines for a chronic condition. Result of this study showed that, in the public sector, the affordability of LPGs was good for most conditions, with standard treatment costs up to 1 days’ income or less. In private sector, the standard treatment of conditions like asthma, diabetes, depression, adult respiratory infection treated with amoxicillin, pediatric respiratory infection, anxiety, pain/ inflammation children and epilepsy treated either by LPGs or by OBs are affordable and cost only 1 days’ income or less of the lowest paid unskilled government worker. However, some conditions like hypertension treated either by bisoprolol or captopril, hypercholesterolemia, arthritis, ulcer treated either by omeprazole or ranitidine, adult respiratory infection treated either by ciprofloxacin or ceftriaxone injection in private sector treated either by LPGs or by OBs need more than 1 day income (1.1–4.4 days) to purchase the needed treatment. This indicates that sometimes when the disease treated either by LPGs or OBs need more than 1 day income. So, this result raises a concern that a patient with low income can’t afford these medicines from private sector. For example, the patient needs to pay up to 4 days’ income for treatment of conditions like ulcer by LPG “omeprazole”. Also, the patient needs to pay up to 4.4 days’ income to afford the treatment of conditions like adult respiratory infection by originator “ciprofloxacin” in private sector. So, patients can afford the prices in public sector more than the prices in private sector.

In general, retailing contributes to additional costs. Rising prices at different stages of the supply chain could lead to higher prices, which would negatively impact the patient’s ability to afford drug costs. So, prices can be reduced if the supply chain is properly organized.

Similar to this result, in a study that was conducted in Haiti, the affordability of treatment for most conditions when treated with LPGs in the public sector were affordable and cost only 1 day income in public sector. Also, in private sector, the conditions were affordable when treated with LPGs costing 1 day wage of the lowest paid government worker. However, when the conditions treated with OBs, prices become less affordable [28]. Another study which was done in Ghana showed that in public sector the average treatment for adult disease conditions with LPGs was not affordable and needed 1.67 days of the income. While, the average treatment for children diseases with LPGs was affordable and needed less than 1 day income (0.78 day) [29].

The findings of this study suggest the implementation of pricing policies to make medicines more affordable and available and to encourage generic prescription, dispensing and substitution. Sustaining the generic policy implementation in the procurement of medicines in the public sector is needed.

### Limitations of the study

There are some limitations to this study. First, the percentage of medicines availability was measured at one time on the day of data collection and may not be the same all year long. Second, the minimum income used for estimation of affordability is that of the governmental sector, where some people do not belong to it. Third, some of medicines that was studied such as metformin and ranitidine were found in different strengths from what were specified from WHO in the medicine price data collection form. Therefore, lower availability of these medicines may not be meaningful, because they were available but in a different strength. Finally, generalizability of affordability result is questionable because affordability was calculated based on the daily wage of the lowest unskilled government worker. However, there may be workers with an income less than the one used in this study.

## Conclusions

In conclusion, this study presented some insight into current issues related to prices, availability and affordability of essential medicines for the treatment of common conditions in Jordan. The study found that in public sector the availability of LPGs was good. However, the availability of OBs was low. The procurement prices of medicines were little bit above the IRPs. Also, the prices of all treatment regimens were reasonable and affordable to the lowest paid unskilled government worker. On the other hand, in private sector, the availability of LPGs and OBs was fairly high. However, the prices of essential medicines were found to be generally high in comparison with IRPs for LPGs as well as OBs and the treatments of most conditions were not affordable. This is likely to persist especially under the current economic situation in Jordan. Pricing policies to reduce the prices in private sector and to ensure that the medicines affordable and available in health sectors should be implemented.

## Additional files

Additional file 1: List of surveyed medicines. A list of 50 medicines was included in this study. The list consisted of global core list of 14 medicines; a regional core list of 16 medicines; and a supplementary list of 20 medicines. (DOCX 17 kb)

Additional file 2: Availability of medicines in public and private sector. The percentage of facility availability of all medicines on the day of data collection. (DOCX 17 kb)

Additional file 3: Median Price Ratios for medicine procurement prices in public sector. Patient prices as MPRs in public sector. (DOCX 15 kb)

Additional file 4: Median Price Ratios for patient prices in public sector. Patient prices as MPRs in public sector. (DOCX 16 kb)

Additional file 5: Median Price Ratios for patient prices in private sector. Patient prices as MPRs in private sector. (DOCX 15 kb)
