# Supplementary material for: Availability, prices and affordability of selected essential medicines in Jordan: a national survey
Source: BMC Health Serv Res. 2018 Oct 19;18:787. doi: 10.1186/s12913-018-3593-9 (PMC6194614; doi:10.1186/s12913-018-3593-9)
Supplement: Supplementary file 1 — List of surveyed medicines. A list of 50 medicines was included in this study. The list consisted of global core list of 14 medicines; a regional core list of 16 medicines; and a supplementary list of 20 medicines. (DOCX 17 kb) [file 12913_2018_3593_MOESM1_ESM.docx]

**Additional file 1**

**List of surveyed medicines**

| **Medicine Name** | **Medicine Strength** | **Dosage Form** | **Target Pack Size** | **Medicine list** |
| --- | --- | --- | --- | --- |
| Amitriptyline | 25 mg | cap/tab | 100 | Global |
| Amoxicillin | 500 mg | cap/tab | 21 | Global |
| Bisoprolol | 5 mg | cap/tab | 60 | Global |
| Captopril | 25 mg | cap/tab | 60 | Global |
| Ceftriaxone injection | 1 g/vial | vial | 1 | Global |
| Ciprofloxacin | 500 mg | cap/tab | 10 | Global |
| Co-trimoxazole | 200+40 mg/ml | suspension | 100 | Global |
| Diazepam | 5 mg | cap/tab | 100 | Global |
| Diclofenac | 50 mg | cap/tab | 100 | Global |
| Metformin | 500 mg | cap/tab | 100 | Global |
| Omeprazole | 20 mg | cap/tab | 30 | Global |
| Paracetamol | 24 mg/ml | suspension | 60 | Global |
| Salbutamol inhaler | 100 mcg/dose | dose | 1 inhaler (200 doses) | Global |
| Simvastatin | 20 mg | cap/tab | 30 | Global |
| Mebendazole | 100mg | cap/tab | 6 | Regional |
| Amoxicillin | 50mg/ml (250mg/5ml) | suspension | 100 ml bottle | Regional |
| Atorvastatin | 20 mg | cap/tab | 30 | Regional |
| Beclometasone | 50 mcg/dose | dose | 200 dose inhaler | Regional |
| Carbamazepine | 200 mg | cap/tab | 100 | Regional |
| Chloramphenicol eye drops | 0.5% | suspension | 5 ml bottle | Regional |
| Dexamethasone injection | 4mg/ml | suspension | 1 ampoule | Regional |
| Fluoxetine | 20 mg | cap/tab | 30 | Regional |
| Furosemide | 40 mg | cap/tab | 30 | Regional |
| Gliclazide | 80 mg | cap/tab | 100 | Regional |
| Ibuprofen | 400 mg | cap/tab | 30 | Regional |
| Lisinopril | 5 mg | cap/tab | 30 | Regional |
| Glibenclamid | 5 mg | cap/tab | 60 | Regional |
| Metronidazole | 500 mg | cap/tab | 14 | Regional |
| Nifedipine Retard | 20 mg | tab | 100 | Regional |
| Ranitidine | 150 mg | cap/tab | 60 | Regional |
| Acyclovir | 400 mg | cap/tab | 25 | Supplementary |
| Enalapril | 20mg | tab | 14 | Supplementary |
| Dilitiazm | 60mg | Tab | 30 | Supplementary |
| Fluconazole | 150mg | Tab | 1 | Supplementary |
| Hydrochlorothiazide | 25mg | cap/tab | 30 | Supplementary |
| Methyldopa | 250mg | tab | 30 | Supplementary |
| Phenytoin | 100mg | cap/tab | 100 | Supplementary |
| Amlodipine | 5mg | tab | 14 | Supplementary |
| Amoxicillin+Clavulanic acid | (500+125) | tab | 20 | Supplementary |
| Doxycycline | 100mg | caps/tab | 10 | Supplementary |
| Allopurinol | 100mg | tab | 100 | Supplementary |
| Azithromycin | 250mgementalhiazide)ee skilled nentered jordan | caps/tab | 6 | Supplementary |
| Isosorbide dinitrate | 5mg | tab | 100 | Supplementary |
| Loratadine | 10mg | tab | 30 | Supplementary |
| Propranolol | 40mg | tab | 50 | Supplementary |
| Metoclopramide HCl | 10mg | tab | 40 | Supplementary |
| Valproic Acid | 200mg | tab | 30 | Supplementary |
| Spironolactone | 25mg | tab | 30 | Supplementary |
| Acetylsalicylic acid | 100mg | tab | 30 | Supplementary |
| Levothyroxin | 0.05 mg | Cap/tab | 100 | Supplementary |
