# Supplementary material for: Availability, prices and affordability of selected essential medicines in Jordan: a national survey
Source: BMC Health Serv Res. 2018 Oct 19;18:787. doi: 10.1186/s12913-018-3593-9 (PMC6194614; doi:10.1186/s12913-018-3593-9)
Supplement: Supplementary file 2 — Availability of medicines in public and private sector. The percentage of facility availability of all medicines on the day of data collection. (DOCX 17 kb) [file 12913_2018_3593_MOESM2_ESM.docx]

**Additional file 2**

**Availability of medicines in public and private sector**

| **Medicine Name** | **Public sector (n =30 outlets )** | | **Private Sector (n=30 outlets )** | |
| --- | --- | --- | --- | --- |
|  | **Originator**  **brand** | **Lowest price**  **generic** | **Originator**  **brand** | **Lowest price**  **generic** |
| Acetylsalicylic acid | 100% | 10% | 93% | 90% |
| Acyclovir | 7% | 73% | 50% | 73% |
| Allopurinol | 0% | 90% | 67% | 87% |
| Amitriptyline | 0% | 40% | 73% | 57% |
| Amlodipine | 0% | 100% | 63% | 73% |
| Amoxicillin | 0% | 100% | 63% | 80% |
| Amoxicillin suspension | 0% | 90% | 70% | 73% |
| Amoxicillin+Clavulanic acid | 0% | 93% | 70% | 87% |
| Atorvastatin | 0% | 83% | 53% | 97% |
| Azithromycin | 0% | 97% | 70% | 100% |
| Beclometasone inhaler | 0% | 87% | 43% | 73% |
| Bisoprolol | 0% | 93% | 80% | 73% |
| Captopril | 0% | 77% | 67% | 87% |
| Carbamazepine | 3% | 87% | 63% | 87% |
| Ceftriaxone injection | 0% | 100% | 47% | 97% |
| Chloramphenicol eye drops | 0% | 93% | 0% | 87% |
| Ciprofloxacin | 0% | 97% | 67% | 87% |
| Co-trimoxazole suspension | 0% | 97% | 53 % | 93% |
| Dexamethasone injection | 0% | 80% | 37% | 90% |
| Diazepam | 33% | 3 % | 60% | 50% |
| Diclofenac Sodium | 0% | 17% | 73% | 77% |
| Dilitiazm | 0% | 77% | 57% | 70% |
| Doxycycline | 0% | 100% | 70% | 93% |
| Enalapril | 0% | 97% | 0% | 87% |
| Fluconazole | 0% | 100% | 67% | 100% |
| Fluoxetine | 0% | 47% | 37% | 70% |
| Furosemide | 0% | 97% | 87% | 80% |
| Glibenclamide | 0% | 100% | 53% | 87% |
| Gliclazide | 0% | 33% | 37% | 57% |
| Hydrochlorothiazide | 0% | 57% | 47% | 57% |
| Ibuprofen | 0% | 97% | 70% | 83% |
| Isosorbide dinitrate | 40% | 57% | 77% | 43% |
| Levothyroxine | 0% | 100% | 83% | 60% |
| Lisinopril | 0% | 7% | 50% | 67% |
| Loratadine | 0% | 93% | 63% | 77% |
| Mebendazole | 90% | 0% | 77% | 86% |
| Metformin | 0% | 7% | 87% | 87% |
| Methyldopa | 3% | 67% | 73% | 43% |
| Metoclopramide | 0% | 100% | 53% | 83% |
| Metronidazole | 0% | 100% | 73% | 83% |
| Nifedipine Retard | 0% | 100% | 0% | 83% |
| Omeprazole | 0% | 100% | 67% | 80% |
| Paracetamol suspension | 0% | 97% | 0% | 93% |
| Phenytoin | 67% | 0% | 83% | 0% |
| Propranolol | 0% | 97% | 0% | 73% |
| Ranitidine | 0% | 33% | 60% | 83% |
| Salbutamol inhaler | 33% | 67% | 67% | 80% |
| Simvastatin | 0% | 73% | 57% | 77% |
| Spironolactone | 0% | 80% | 60% | 63% |
| Valproic Acid | 100% | 0% | 60% | 53% |
