# Supplementary material for: Availability, prices and affordability of selected essential medicines in Jordan: a national survey
Source: BMC Health Serv Res. 2018 Oct 19;18:787. doi: 10.1186/s12913-018-3593-9 (PMC6194614; doi:10.1186/s12913-018-3593-9)
Supplement: Supplementary file 4 — Median Price Ratios for patient prices in public sector. Patient prices as MPRs in public sector. (DOCX 16 kb) [file 12913_2018_3593_MOESM4_ESM.docx]

**Additional file 4**

**Median Price Ratios for patient prices in public sector**

| **Medicine Name** | **Originator brand MPR** | **Lowest price generic MPR** |
| --- | --- | --- |
| Acetylsalicylic acid |  |  |
| Acyclovir |  | 1.43 |
| Allopurinol |  | 0.49 |
| Amitriptyline |  | 3.34 |
| Amlodipine |  | 0.28 |
| Amoxicillin |  | 1.05 |
| Amoxicillin suspension |  | 1.15 |
| Amoxicillin+Clavulanic acid |  | 0.49 |
| Atorvastatin |  | 0.46 |
| Azithromycin |  | 0.59 |
| Beclometasone inhaler |  | 0.78 |
| Bisoprolol |  | 0.26 |
| Captopril |  | 0.71 |
| Carbamazepine |  | 1.17 |
| Ceftriaxone injection |  | 0.91 |
| Chloramphenicol eye drops |  | 1.19 |
| Ciprofloxacin |  | 0.72 |
| Co-trimoxazole suspension |  | 0.06 |
| Dexamethasone injection |  | 1.28 |
| Diazepam |  |  |
| Diclofenac Sodium |  | 1.56 |
| Dilitiazm |  | 0.35 |
| Doxycycline |  | 1.81 |
| Enalapril |  | 2.45 |
| Fluconazole |  | 1.43 |
| Fluoxetine |  | 1.18 |
| Furosemide |  | 4.02 |
| Glibenclamide |  | 1.25 |
| Gliclazide |  | 0.82 |
| Hydrochlorothiazide |  | 4.28 |
| Ibuprofen |  | 1.80 |
| Isosorbide dinitrate |  | 0.50 |
| Levothyroxine |  | 1.23 |
| Lisinopril |  |  |
| Loratadine |  | 0.14 |
| Mebendazole | 8.26 |  |
| Metformin |  |  |
| Methyldopa |  | 1.31 |
| Metoclopramide |  | 3.82 |
| Metronidazole |  | 1.87 |
| Nifedipine Retard |  | 0.40 |
| Omeprazole |  | 1.57 |
| Paracetamol suspension |  | 1.26 |
| Phenytoin | 5.81 |  |
| Propranolol |  | 5.29 |
| Ranitidine |  |  |
| Salbutamol inhaler |  | 1.13 |
| Simvastatin |  | 0.45 |
| Spironolactone |  | 0.69 |
| Valproic Acid | 0.56 |  |

MPR: Median Price Ratio
