# Supplementary material for: Availability, prices and affordability of selected essential medicines in Jordan: a national survey
Source: BMC Health Serv Res. 2018 Oct 19;18:787. doi: 10.1186/s12913-018-3593-9 (PMC6194614; doi:10.1186/s12913-018-3593-9)
Supplement: Supplementary file 5 — Median Price Ratios for patient prices in private sector. Patient prices as MPRs in private sector. (DOCX 15 kb) [file 12913_2018_3593_MOESM5_ESM.docx]

**Additional file 5**

**Median Price Ratios for patient prices in private sector**

| **Medicine Name** | **Originator brand MPR** | **Lowest price generic MPR** |
| --- | --- | --- |
| Acetylsalicylic acid | 47.94 | 16.64 |
| Acyclovir | 5.51 | 11.45 |
| Allopurinol | 0.78 | 1.14 |
| Amitriptyline | 12.12 | 6.16 |
| Amlodipine | 12.14 | 13.65 |
| Amoxicillin | 9.75 | 7.54 |
| Amoxicillin suspension | 5.25 | 5.33 |
| Amoxicillin+Clavulanic acid | 2.95 | 1.78 |
| Atorvastatin | 28.84 | 22.09 |
| Azithromycin | 22.53 | 14.57 |
| Beclometasone inhaler | 1.23 | 1.07 |
| Bisoprolol | 3.23 | 3.38 |
| Captopril | 22.42 | 15.89 |
| Carbamazepine | 6.34 | 4.93 |
| Ceftriaxone injection | 36.4 | 24.06 |
| Chloramphenicol eye drops |  | 1.77 |
| Ciprofloxacin | 59.88 | 18.84 |
| Co-trimoxazole suspension | 6.23 | 3.4 |
| Dexamethasone injection | 9.81 | 8.53 |
| Diazepam | 5.98 | 23.78 |
| Diclofenac Sodium | 29.91 | 23.04 |
| Dilitiazm | 1.85 | 1.48 |
| Doxycycline | 58.36 | 27.12 |
| Enalapril |  | 47.52 |
| Fluconazole | 146.99 | 73.85 |
| Fluoxetine | 35.49 | 28.46 |
| Furosemide | 10.67 | 11.27 |
| Glibenclamide | 19.89 | 11.25 |
| Gliclazide | 3.87 | 2.74 |
| Hydrochlorothiazide | 29.81 | 30.30 |
| Ibuprofen | 6.44 | 7.2 |
| Isosorbide dinitrate | 1.00 | 1.24 |
| Levothyroxine | 2.20 | 3.25 |
| Lisinopril | 2.64 | 2.92 |
| Loratadine | 10.16 | 7.6 |
| Mebendazole | 51.68 | 37.05 |
| Metformin | 3.87 | 3.22 |
| Methyldopa | 3.38 | 2.11 |
| Metoclopramide | 13.49 | 4.63 |
| Metronidazole | 28.34 | 9.03 |
| Nifedipine Retard |  | 8.72 |
| Omeprazole | 29.73 | 61.37 |
| Paracetamol suspension |  | 1.49 |
| Phenytoin | 9.74 |  |
| Propranolol |  | 6.34 |
| Ranitidine | 5.58 | 4.47 |
| Salbutamol inhaler | 2.35 | 1.23 |
| Simvastatin | 7.84 | 6.27 |
| Spironolactone | 3.97 | 3.01 |
| Valproic Acid | 0.64 | 0.81 |

MPR: Median Price Ratio
